# Supplementary figures and images for: Systematic Review of Lung Cancer Screening: Advancements and Strategies for Implementation
Source: Healthcare (Basel). 2023 Jul 21;11(14):2085. doi: 10.3390/healthcare11142085 (PMC10379173; doi:10.3390/healthcare11142085)

# Supplementary material

## Number of included studies with timeline

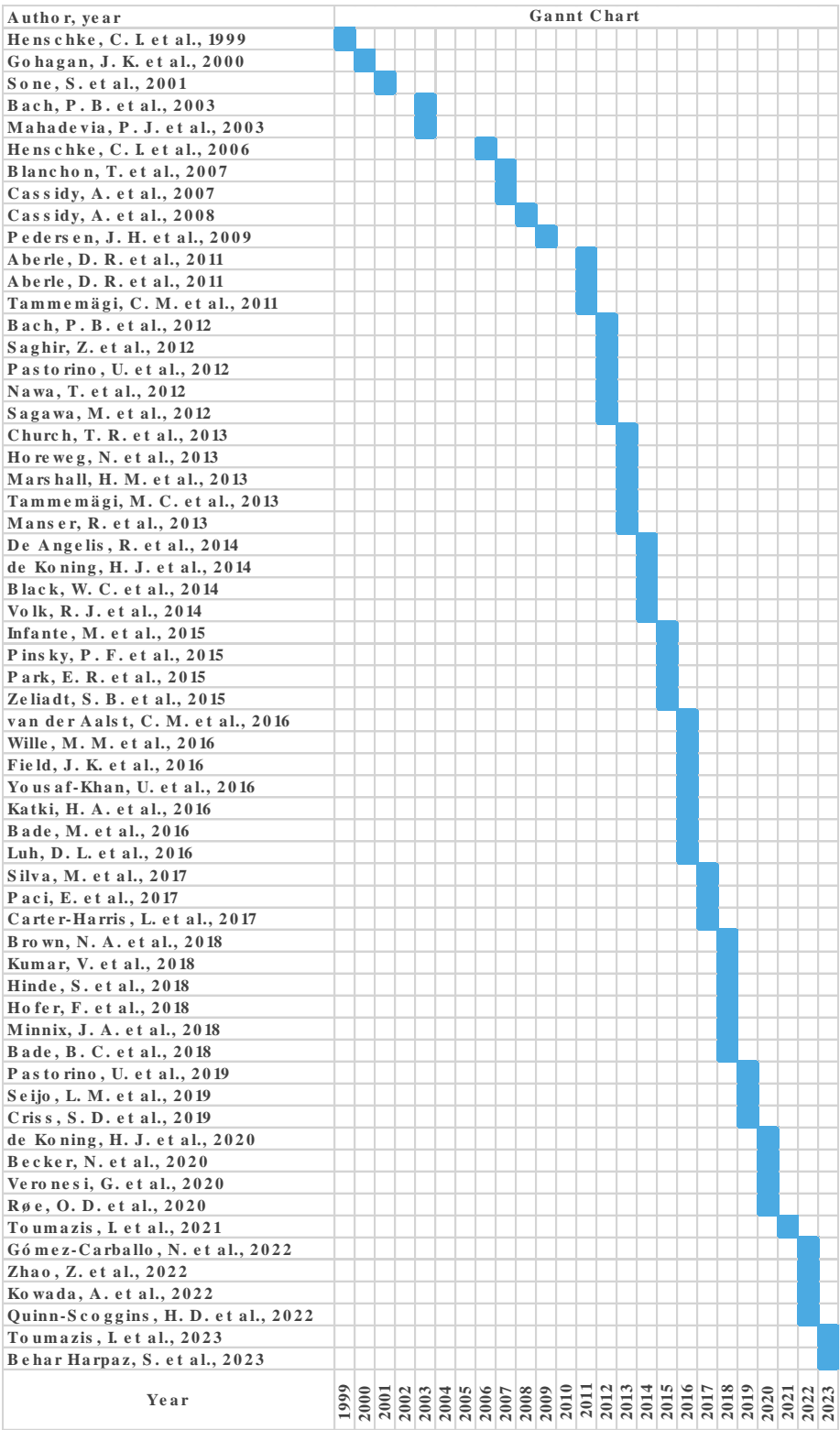

Supplement: Supplementary file 1 [file healthcare-11-02085-s001.zip › healthcare-2476709-supplementary.pdf]
